# The effect of progressive image scrambling on neuronal responses at three stations of the pigeon tectofugal pathway

**DOI:** 10.1038/s41598-022-18006-0

**Published:** 2022-08-19

**Authors:** William Clark, Matthew Chilcott, Michael Colombo

**Affiliations:** 1 Neural Basis of Learning, Institute of Cognitive Neuroscience, Faculty of Psychology, Ruhr-University Bochum, Bochum, Germany; 2 Department of Physics, University of Otago, Dunedin, New Zealand; 3 Department of Psychology, University of Otago, Dunedin, New Zealand

**Keywords:** Visual system, Neuroscience, Sensory processing

## Abstract

The progressive image scrambling procedure is an effective way of determining sensitivity to image features at different stages of the visual system, but it hasn’t yet been used to evaluate neuronal responses in birds. We determined the effect of progressively scrambling images of objects on the population responses of anterior entopallium (ENTO), mesopallium ventrolaterale (MVL), and posterior nidopallium intermediate pars lateralis (NIL) in pigeons. We found that MVL responses were more sensitive to both the intact objects and the highly scrambled images, whereas ENTO showed no clear preference for the different stimuli. In contrast, the NIL population response strongly preferred the original images over the scrambled images. These findings suggest that the anterior tectofugal pathway may process local shape in a hierarchical manner, and the posterior tectofugal pathway may process global shape of greater complexity. Another possibility is that the differential responses between ENTO/MVL and NIL may reflect an anterior–posterior map of varying sensitivity to spatial frequency.

## Introduction

The avian visual system is organised in layered columns like the mammalian cerebral cortex^1–3^. Recent investigation of the auditory^4,5^ and visual pathways^6,7^ also suggests that these layers process information in a hierarchal manner, with a progressive increase in complexity of the sensory information represented at subsequent stages. While the hierarchy between layers are believed to perform computations that distinguish between different object categories, even when unbounded by low-level similarity^8^, we lack a detailed account of how the avian visual system produces these representations in comparison with primates and rodents.

A technique that has been used to map the selectivity of neurons at different stages of the mammalian visual system to image features is the progressive image scrambling procedure. The technique compares neuronal activity in response to object stimuli with scrambled versions of the objects, where a progressively increasing number of square segments are randomly rearranged. The progressive image scrambling procedure was used to determine, using fMRI imaging, what degree different human^9,10^ and macaque^11^ visual cortical areas represent whole objects as opposed to their parts. These studies found that extrastriate cortex displayed similar activation to intact objects and scrambled images up to very high scrambling levels, consistent with intermediate shape processing complexity. Inferior-temporal (IT) cortex displayed monotonic decreases in activity to increasing scrambling levels, consistent with greater global object feature analysis and larger receptive fields than at the level of earlier ventral stream regions. A comparable preference for natural movies over scrambling manipulations^12^ and increasingly non-linear shape computations^13^ has also been revealed along the rat ventral stream.

In contrast with higher ventral stream stages, V1 of humans^9,10^, macaques^11^, marmoset^14^, and rats^12^ displays particularly strong activation in response to scrambled images. The strong responses to scrambled images in mammalian early visual cortex^11^ and the early layers of computational models of object recognition^15^ correlates with an increase in the average power of the high spatial frequency band resulting from the introduction of additional edges into the images.

Despite the fact that progressive image scrambling can indicate differences in visual feature processing between early and higher visual structures in mammalian brain, no study has assessed the effect of progressive image scrambling on neuronal responses in birds. We examined the neuronal responses of three areas in the avian tectofugal system, that visual pathway used mainly for processing objects at close distance, such as would be encountered in a behavioural chamber. The three regions consisted of the anterior entopallium (ENTO), the mesopallium ventrolaterale (MVL), and the nidopallium intermediate pars lateralis (NIL). The anterior ENTO forms a topographic connection with the dorsally positioned MVL layer, while posterior ENTO projects primarily to the NIL^1,16,17^. A recent study determined that an MVL population responded more strongly to images of objects and one level of scrambling in comparison with an ENTO population^7^, similar to the responses observed in the mammalian early visual cortex to these types of stimuli. The aim of the present study was to build on these findings and determine if a neuronal population isolated from ENTO, MVL and NIL displayed differences in response selectivity for images of objects scrambled at progressively increasing levels.

## Results

### Single-unit analysis of ENTO, MVL and NIL responses

**Figure 1:**
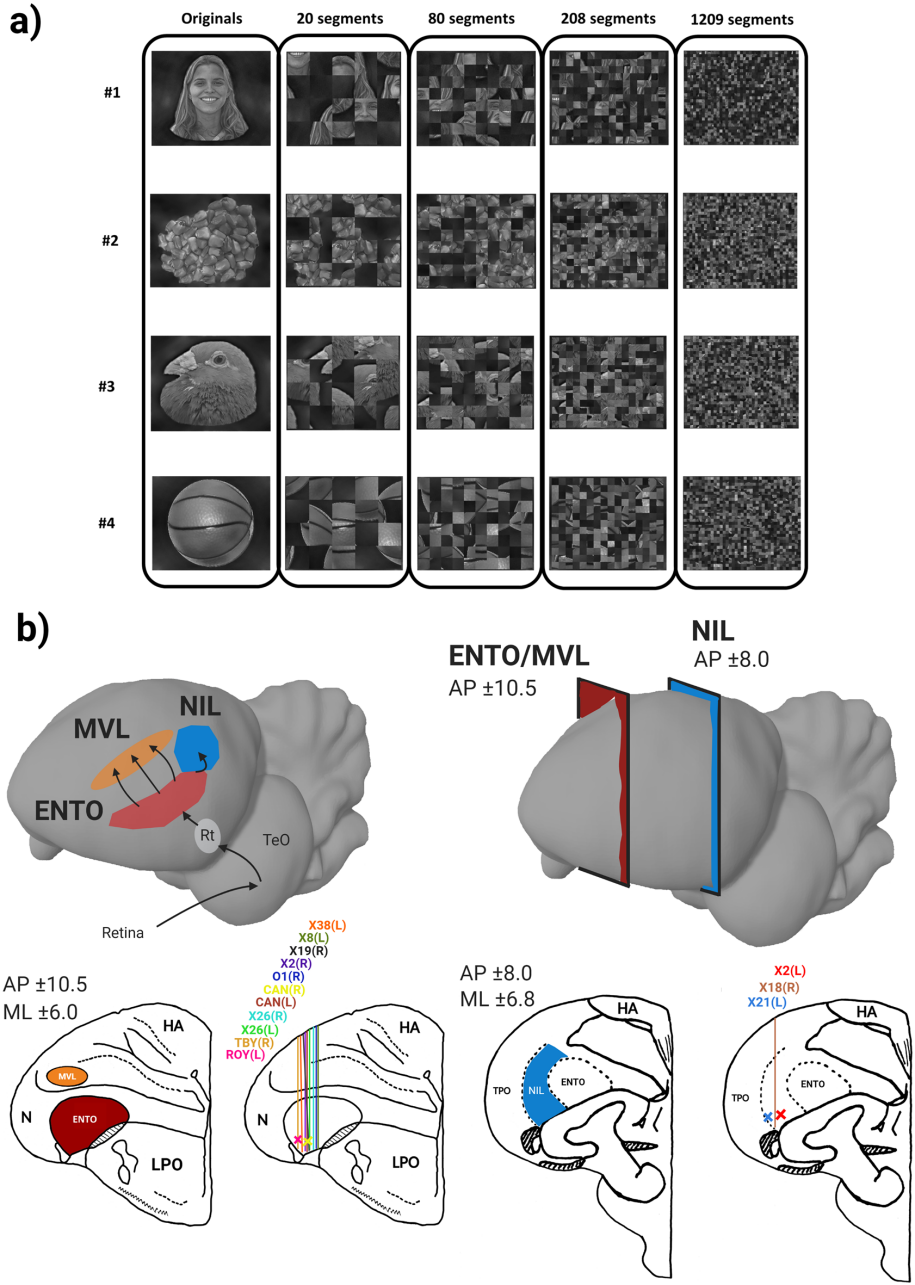
(**a**) Stimulus set used for the experiment. Images of four different objects (originals) and four corresponding levels of progressive image scrambling (20, 80, 208, and 1209 segments). (**b**) Targeted MVL (orange), ENTO (red), and NIL (blue) regions. Also depicted is the electrode track reconstruction for all pigeons at the corresponding AP ± 10.5 and ± 8.0 coronal brain sections. Abbreviations^16^: nidopallium (N), nidopallium intermediate pars lateralis (NIL), mesopallium ventrolaterale (MVL), hyperpallium apiciale (HA), lobus parolfactorius (LPO), area temporo-parieto-occipitalis (TPO).

**Figure 2:**
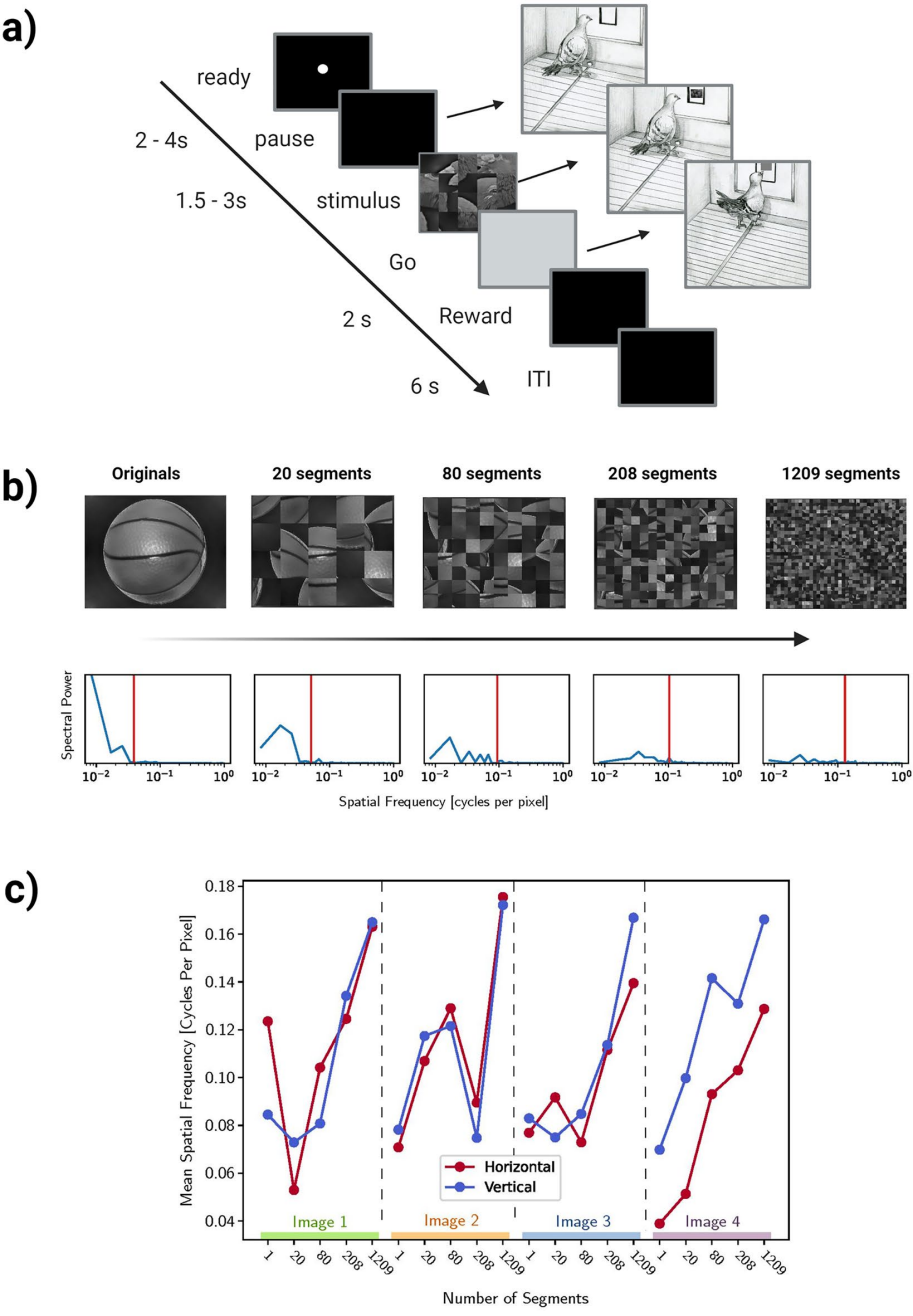
(**a**) A typical experimental trial started with two pecks to the orienting dot stimulus, initiating a pause period between 2 and 4 s with a black screen. An image was displayed in the response window for a random time (1.5–3 s) in the stimulus period. Pecks at the image started a correction repeat of the same trial from the start of the ITI. If the bird withheld pecks during the stimulus period, a Go cue (grey square) replaced the image on screen, and a peck to the Go cue delivered grain reward from the hopper, paired with a light and tone. A 6 s ITI followed the reward period. (**b**) Depiction of the progressive scrambling procedure for the original images (top) and corresponding spectral density plots (bottom). Low spatial frequencies are disrupted, and the average energy across spatial frequency bands increases (particularly high spatial frequencies) as the images become progressively more scrambled. The mean horizontal spatial frequency is highlighted by the red line, serving as an effective proxy for the changes in the distribution of spatial frequency components. (**c**) Spectral analysis of the image set showed that the progressive scrambling procedure generally resulted in an increase of high spatial frequency information across the horizontal and vertical directions at increasing levels of scrambling for each of the four original images.

We examined the responses of neurons in ENTO, MVL and NIL to images of objects and four levels of progressive scrambling (Fig. 1a,b) during a response inhibition task used previously to map selectivity to visual stimuli in the pigeon visual system^7^. The task required that the pigeons inhibited pecking responses and viewed the images displayed on screen for a randomly determined time (1.5–3 s) to obtain grain reward, without needing to behaviourally categorise them (Fig. 2a).

A paired *t*-test (*p* < 0.05) was used to compare each neuron’s firing rates in a 500 ms window after the onset of the stimulus with a 500 ms window in the baseline intertrial interval (ITI) period. In ENTO we recorded from a total of 56 neurons of which 34 (61%) were visually responsive. In MVL we recorded from a total of 96 neurons of which 54 (56%) were visually responsive. In NIL we recorded from a total of 62 neurons of which 44 (71%) were visually responsive. The proportion of visually-responsive neurons was similar between the three regions (χ^2^ (2) = 3.48, *p* = 0.17). In all three regions we encountered more excitatory visually-responsive neurons (relative to baseline) compared to inhibitory visually-responsive neurons (ENTO: 76% vs. 24%; MVL: 80% vs. 20%; NIL: 89% vs. 11%). All three regions displayed similar proportions of excitatory and inhibitory neurons (χ^2^ (2) = 2.2, *p* = 0.33).

**Figure 3:**
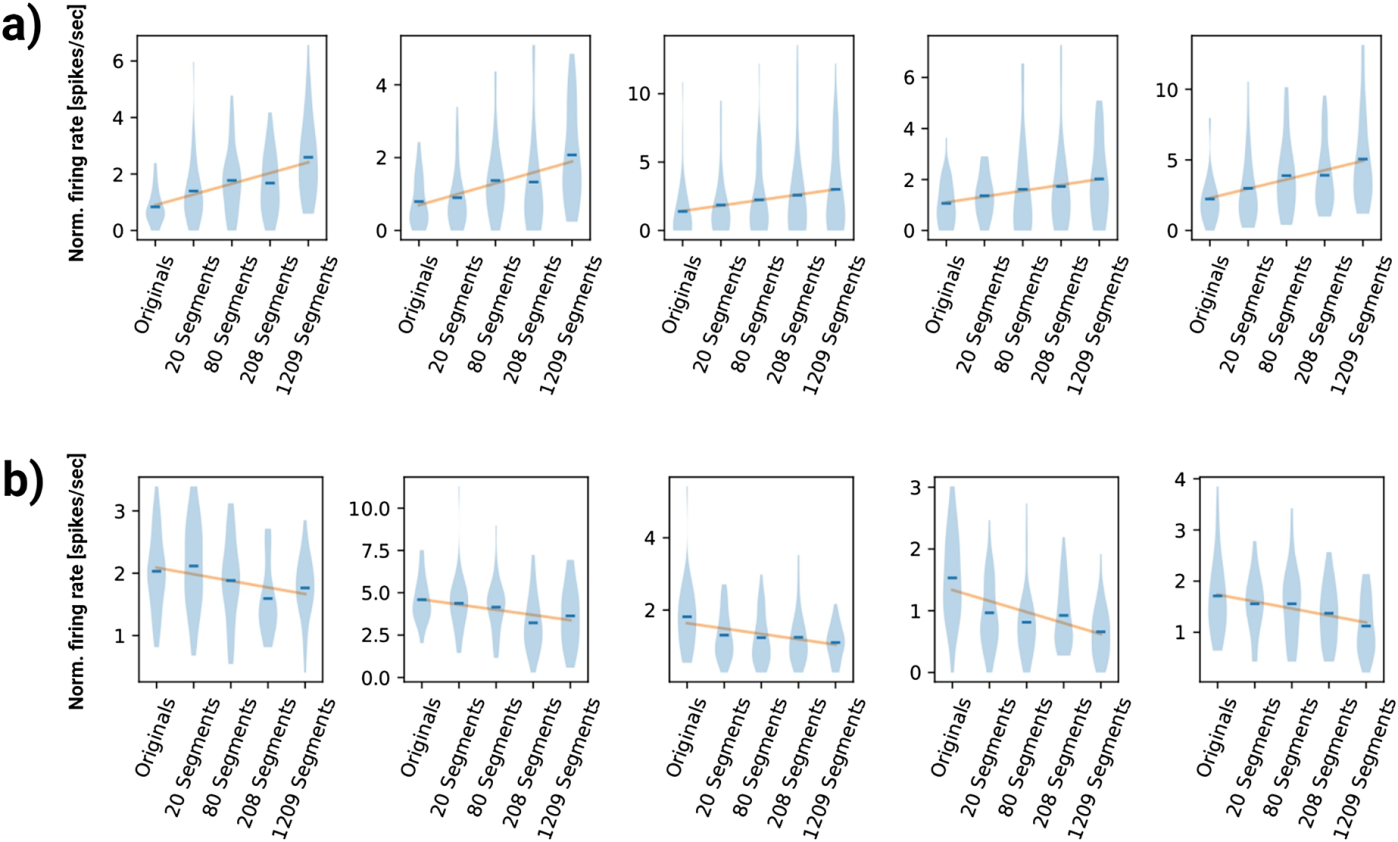
(**a**) Five example scrambling-responsive neurons that responded best to increasing levels of scrambling. (**b**) Five example scrambling-responsive neurons that fired maximally to the original images of objects and decreased responses to scrambling.

We next determined the capacity of the scrambling manipulations (Fig. 2b,c) to explain neuronal responses. We found individual neurons across all three regions that displayed monotonic tuning to either the highest level of scrambling (Fig. 3a) or the original objects (Fig. 3b), with stepwise increases or decreases in firing rates based on the level of progressive image scrambling. Other neurons were most responsive to the mid-levels of image scrambling, or displayed no clear preference for the scrambling manipulations compared to the original objects. We next examined how many of the visually-responsive neurons between the three regions displayed a significant difference (one-way ANOVA, *p* < 0.05) in firing rates between the different scrambling levels in the stimulus period. In ENTO, 4 (12%) of the 34 visually responsive neurons were scrambling-responsive. In MVL, 7 (13%) of the 54 visually-responsive neurons were scrambling responsive. In NIL, 13 (30%) of the 44 visually responsive neurons responded significantly to scrambling. There was a significantly greater proportion of scrambling-responsive neurons in NIL relative to MVL (χ^2^ (1) = 4.1, *p* = 0.042), and a trend towards more scrambling-responsive neurons in NIL relative to ENTO (χ^2^ (1) = 3.55, *p* = 0.059).

### Bayesian linear regression reveals differences in image feature sensitivity between ENTO, MVL and NIL

**Figure 4:**
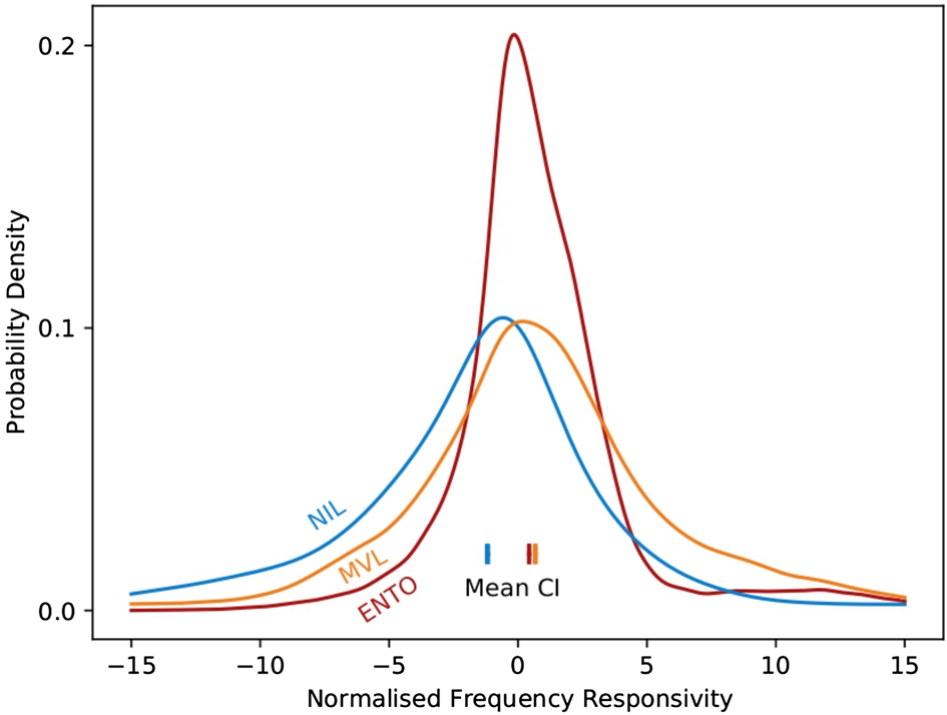
Posterior density distributions of response gradients resulting from the Bayesian GLM for ENTO, MVL and NIL. A large proportion of neurons in MVL and ENTO display a non-zero gradient, with a dependence on scrambling in the positive direction. In contrast a large proportion of neurons in NIL display a dependence on scrambling in the negative direction. Bayesian estimation tests^18^ revealed significant differences between means (coloured at bottom) of the distributions of firing-rate vs. scrambling level slopes between all the three regions.

The stepwise responses of many single neurons in ENTO, MVL and NIL to objects or the scrambled images suggests that all three regions carry information that can distinguish between the features of the images. If these regions are part of separate stages of the sensory processing chain in the avian brain^4–7^, the population response of the three regions might display differences in sensitivity to the scrambling manipulations. We determined whether the single-trial responses of all the visually responsive neurons isolated from ENTO, MVL and NIL displayed differences in their population response gradients to the objects’ global features, or the scrambled images’ local features, using a Bayesian general linear model (Fig. 4). We used Bayesian estimation tests^18^ to compare the posterior density distributions of the firing rate versus image scrambling level linear fits for the ENTO, MVL and NIL responses resulting from the Markov-chain Monte-Carlo sampling. Bayesian estimation tests compare the relative credible intervals (CI) of every possible difference of means, standard deviations, and effect sizes.

The estimated mean slopes of ENTO (0.43, *ν* = 2), MVL (0.67, *ν* = 2), and NIL (− 1.19, *v* = 4) were are all significantly different from each other, and from zero. Likewise, the estimated standard deviations of ENTO, (± 1.70), MVL (± 3.37), and NIL (± 3.67) also displayed significant differences from each other, and from zero. These analyses revealed that the image features that were altered by progressive scrambling were represented differently by a neuronal ensemble in ENTO, MVL and NIL. The CI’s of the posterior density distributions of all three regions differed from the expected Gaussian distribution of slopes at chance level, with MVL and NIL displaying a high proportion of samples falling above the tails with strong response gradients.

The posterior density distributions are shown in Fig. 4. In addition, ENTO had the largest degree of posterior density samples centred around zero compared with the other regions, indicating that many cells displayed sparse tuning to the different stimuli and little variation in responses to the scrambling manipulations. The bulk of the MVL population's distribution of response gradients favoured positive gradients, but also displayed a higher proportion of samples at the tail end that preferred strong negative gradients compared with ENTO. The strong rightward shift of the MVL posterior distribution suggests that the population response is most dependant on the increasing degree of progressive image scrambling. The preference for some strong negative slopes at the tail of the MVL posterior distribution suggests that the population also represents features found in the images of the original objects that are disrupted by scrambling. The means of the CI’s for ENTO and MVL showed the closest similarity, centred at marginally positive slopes relative to zero. In contrast, the NIL populations distribution of response gradients was shifted substantially leftward, with the highest degree of samples showing strong negative response gradients. Moreover, very little of the NIL slope samples had positive gradients, especially relative to the MVL distribution's positive tail. The mean CI for NIL was shifted highly negative relative to zero, displaying a much larger difference from the mean CI’s of ENTO and MVL. These characteristics of the posterior density distribution suggests that the NIL population response is most dependant on the features of the intact original objects that are disrupted by progressive image scrambling.

## Discussion

The present study investigated the effect of progressive image scrambling on the responses of ENTO, MVL, and NIL neurons, three visual processing stations of the avian tectofugal system. The neuronal population response gradients to the scrambling manipulations displayed substantial differences between all three regions. MVL responses were mostly skewed towards positive with some negative slopes in the tails with increasing scrambling levels, whereas the ENTO population showed sparse tuning to the original objects and scrambling manipulations. In contrast, the NIL population response was shifted strongly towards negative slopes for increasing scrambling levels, preferring the original images over the scrambling manipulations.

### Anterior tectofugal regions ENTO and MVL process different image information

The substantial increase in the selectivity of the MVL population to the progressive scrambling manipulations relative to ENTO confirms and extends the findings of Clark et al.^7^, who found a stronger response to both images of objects and one level of scrambling at the level of MVL in comparison with ENTO. Scrambling causes two major changes of activity in the mammalian visual cortex^9–11^. The first effect is a reduction in the activity of shape-selective neurons which are sensitive to the configuration of global stimulus features. The second effect of scrambling is the strong excitation of neuronal populations in the early visual system, which is associated with changes in the images spatial frequency content.

The strong responses of many MVL neurons to intact objects is consistent with the notion that the anterior tectofugal pathway layers are involved in shape processing^19,20^, and that image features may be hierarchically recoded between ENTO and MVL to support more complex feature identification^6,7^. While some MVL cells responded preferentially to the images of intact objects, many neurons also monotonically increased their responses to the greater levels of scrambling. The strong influence of increasing scrambling levels on the population response of MVL is very similar to the effects typically observed in early visual cortex^9–12^. These findings are different from higher stages of the primate and rodent ventral stream beyond V1, where the feed-forward organisation across the hierarchy of regions results in progressively stronger responses to intact objects compared with scrambled stimuli^9–12,21^.

### NIL in the posterior tectofugal pathway represents different image information compared to the anterior tectofugal pathway

In contrast with the anterior regions, the population response of the posteriorly located NIL preferred the original images of objects over the increasing scrambling levels. There are two possible reasons that can explain NIL’s sensitivity to the unscrambled objects. The first explanation is that NIL is further along the putative hierarchy of visual areas in the pigeon brain, and neurons display selectivity for configurational object shape that is disrupted by the scrambling process, as seen in higher-order ventral stream stages^9–12,21^. We believe that this scenario is unlikely as the number of scrambling-responsive single neurons that fired selectively to the objects and decreased their responses progressively was low in NIL (4/44 visually responsive neurons: 9%) relative to IT cortex (35/40 visually responsive neurons: 88%^21^).

The second potential explanation for the preference of the population response for images of objects, is that the NIL neurons are sensitive to the general low spatial frequency content shared across the images depicting objects (see Fig. 2c), as opposed to their global shape. A gradient of anterior–posterior sensitivity to spatial frequency may provide an explanation for the strong selectivity in response to scrambled images observed in MVL within the anterior tectofugal pathway, and the strong preference for original objects observed in NIL within the posterior tectofugal pathway. To the best of our knowledge, these are the first single-unit recordings that have been performed from NIL to detail its response properties to images. At present, there is very little available evidence that can provide clues to NIL’s role in vision. NIL receives anatomical projections primarily from posterior ENTO^17^, which is known to be engaged primarily in motion perception, as opposed to shape processing^19,20^. However, while previous ZENK imaging studies implicate NIL with a role in analysing visual signals related to live conspecifics^22^, another study found that NIL displayed no differences in activation between form/color and motion perception tasks relative to a control condition^23^.

### Progressive image scrambling makes it difficult to dissociate shape and spatial frequency explanations of neuronal activity

The present study highlights some important limitations of the progressive scrambling procedure that are important to bear in mind when investigating object processing. Firstly, as the image statistics change dramatically when images are scrambled, it’s not clear if the difference in activity at the level of NIL is because of the manipulation of an object's global shape, or simply because of different information being fed forward from regions lower in the visual hierarchy^15^. The second issue is that low spatial frequencies and global shape properties are also confounded, as are high spatial frequencies and the local edges introduced by the scrambling procedure used to modify the original images. Band-pass image filtering techniques^24–27^ should be used in future studies to dissociate between global shape, and low spatial frequency image content as explanatory variables of stronger neuronal activity to intact objects relative to control stimuli.

## Conclusion

We found evidence that an MVL neuronal population responds strongly to both original objects and scrambled images, and does so with greater capacity to distinguish between different scrambling levels than at the level of ENTO. These observations indicate that MVL is located at a later hierarchical stage of processing, yet many local image features typical of the early visual cortex are processed at this station as well. In contrast, a neuronal population from the posterior brain region NIL responded best to images of intact objects. The preference for intact objects displayed by NIL may be related to a role in global shape processing, but may also be related to the lower spatial frequency content of the intact object images. These findings suggest that the anterior and posterior tectofugal pathway may process qualitatively different types of visual information.

## Methods

### Subjects

Ten experimentally naive pigeons (*Columba livia*) served as experimental subjects (CAN, ROY, O1, X2, X8, X18, X19, X21, X26, and X38). Each pigeon was housed in wire mesh cages individually, and the colony room was maintained at 20 °C. The birds were fed a blend of wheat, peas, and corn, and had ad libitum access to grit and water. The pigeons were maintained at 85% of their free feeding weight during the experiment. All experimental procedures were approved by the University of Otago Animal Ethics Committee and conducted in accordance with the University of Otago’s Code of Ethical Conduct for the Manipulation of Animals and the ARRIVE guidelines for the care and use of laboratory animals.

### Apparatus

Pigeons were trained and tested using standard operant chambers with dimensions of 32.5 cm (length), 36 cm (width) and 34.5 cm (height). A 17-inch screen (resolution: 1284 × 1024) was used to present stimuli. A Carroll Touch infrared touch frame (EloTouch, baud rate 9600, transmission time 20 ms) in front of the screen registered the XY coordinates of pecks. A transparent plexiglass panel was placed in front of the screen and had a single square response key (2.5 × 2.5 cm), to prevent accidental responses from being registered. Grain reward was delivered via a food hopper 20 cm below the square response key, which was illuminated when raised.

### Stimuli

Twenty images were used as visual stimuli (Fig. 1a), consisting of four images of different objects serving as the base images (Original) and four levels of scrambling of the base images with a progressive increase in the number of scrambled segments (20, 80, 208, and 1209 segments). The four original objects depicted are a human face, grain, pigeon face, and a basketball. The human face image was obtained and modified from the FEI face database available at (https://fei.edu.br/~cet/facedatabase.html). The images of the pigeon face, grain and basketball were taken using a Cannon DS126291 digital camera. A critical step to determine the effect of progressive image scrambling is to match any pre-existing differences in low-level features between the images of the four original objects. We matched the images low-level properties by equalising the rotational average of the Fourier amplitude spectra at each spatial frequency using the SHINE toolbox in MATLAB^28^. Once the Fourier amplitude spectra was equated between the four original images, four levels of progressive image scrambling were generated by randomly shuffling the position and orientation of the tiles.

### Behavioural task

We utilised the same response inhibition task as in our previous investigation of the pigeon visual system^7^. We first trained pigeons to respond with a peck to a white dot to receive a grain reward. Next they were trained to withhold responses while a visual stimulus was displayed on the screen. The procedure on a typical trial was as follows (Fig. 2a). A white dot was displayed in the centre of the response key during the ready period. Any pecks during the pause period extended the pause period by 2 s. Two pecks to the white dot turned it off and started a pause period of a random time between 2 and 4 s. Any pecks in the first 0.5 s of the pause period were ignored to prevent pecks directed towards the ready stimulus from extending the duration of the pause period. Following the pause period, a stimulus period started during which an image was displayed in the response window for a random time between 1.5 to 3 s. Pecks in the stimulus period immediately turned off the stimulus and initiated a correction repeat of the same trial from the start of the intertrial interval (ITI) period. Following the stimulus period, a Go cue (grey square) appeared in place of the stimulus, letting the bird know that it was required to respond with a single peck to the Go cue. A peck to the Go cue turned it off and started the reward period with access to grain from the hopper for 1.75 s duration, accompanied by a 1000-Hz tone and the illumination of the hopper. After the delivery of reward, an ITI of 6 s started, with any pecks during the ITI extending its duration by 2 s.

### Surgery

Once the pigeons had achieved reliable performance on the task, we installed a movable microdrive into the target brain areas via a stereotaxic surgery. An anaesthetic mixture of Ketamine (30 mg/kg) and Xylazine (6 mg/kg) was injected into the legs. The feathers on the head were removed, and the head was immobilised in a Revzin stereotaxic adapter^29^. A topical anaesthetic (10% Xylocaine) was applied to the scalp and the skin overlying the skull was retracted, exposing the skull. Six stainless steel screws were inserted into the skull, with one of the screws serving as the ground screw. A hole was drilled above the targeted area and the dura removed. Eight pigeons (ROY, O1, CAN, X2, X8, X19, X26, and X38) had microdrives installed at positions AP ± 10.5 mm, and ML ± 6.0 mm, corresponding to the location of anterior MVL, and below it, ENTO. Three pigeons (X2, X18, and X21) had microdrives installed at positions AP ± 8.0 mm, and ML ± 6.8 mm, corresponding to the location of NIL. Note that two of the ten total birds received implants in both the left and right hemisphere of ENTO (CAN and X26; Fig. 1b), and one bird received an implant in both the left hemisphere of NIL and right hemisphere of ENTO (X2; Fig. 1b) to increase the total yield of neurons.

### Neuronal recording

The microdrives contained eight 25 μm Formvar-coated nichrome wires (California Fine Wire, Grover Beach, CA, USA) used to measure single neuron activity^30^.We searched for activity on any one of the eight wires and used one of the remaining wires as the indifferent. The signals were amplified using a Grass P511K amplifier (Grass Instruments, Quincy, MA, USA) and 50 Hz noise was eliminated using a notch filter. A CED (Cambridge Electronic Design, Cambridge, UK) electrophysiology system with Spike2 software stored and analysed the data. Cells were isolated using CED’s template matching capacity (thereby eliminating artefacts) sampling at a rate of 20,000 Hz. The selection criterion was that the isolated neuron had a signal-to-noise ratio of no less than 2:1. A separate computer controlled the behavioural task and sent codes to the CED system to align key task events. The electrodes were advanced approximately 40 μm after each recording session before the pigeon was returned to their home cage. If we did not record from any neural activity the electrodes were moved approximately 20 μm, and the animal was returned to its cage.

For the birds that were implanted in MVL (X8, X19, X26, and X38), we advanced the electrode through the entire extent of MVL (2000 μm), then the electrode was advanced another 500 μm into ENTO. We then recorded activity from units in the entire extent of ENTO (3000 μm) directly ventral to MVL. Another four birds received implants directly in ENTO (X2, CAN, O1, and ROY) to balance the numbers of recorded neurons. For the three birds that were implanted in NIL (X2, X18, and X21) we advanced the electrode through the full NIL region (4800 μm). Recording sessions took approximately 1 h to complete and pigeons performed one session daily for 5 days a week.

### Histology and electrode track reconstruction

Following the end of recording sessions, a 9 V potential was sent through each electrode for 10 s to create an electrolytic lesion marking the recording position of each electrode. Pigeons were then euthanized via carbon dioxide gas, and were perfused with physiological saline and 10% formalin. The brains were removed from the skull and kept in 10% formalin for at least 5 days, followed by sucrose formalin (10% formalin, 30% sucrose). The brains were frozen and sliced into 40 µm sections and stained with thionin. Track reconstructions were made using the position of the electrolytic lesion and depth records. All of the recovered electrode tracks were within 0.5 mm of the borders of the targeted ENTO, MVL and NIL regions^29^ (see Fig. 1b). For several pigeons, we were unable to recover the electrode track positions (ROY, CAN X21, X22) and the final termination points were estimated using the co-ordinates of the visible craniotomy entry point during blocking, and the electrode depth records.

### Single-unit analysis

Each neurons spiking data for behavioural sessions was loaded into MATLAB (version 2016b) for data analysis. Neurons were required to exhibit baseline mean firing rates > 0.2 Hz during the ITI period (middle 500 ms of the ITI) to be included in the analysis. We used the firing rate data over all 160 trials that the bird successfully inhibited responses to images until the grey square appeared; and we discarded correction trials data from the analysis. Neurons were classified as visually responsive if the firing rate over the 160 trials in the stimulus period (500 ms post stimulus onset) was significantly greater (paired *t*-test (159), two tailed: *p* < 0.05) than the neurons’ firing rate during the baseline ITI period (500 ms). The response was defined as ‘excitatory’ if the average response in the stimulus period was greater than the average baseline ITI response. The response was classified as ‘inhibitory’ if the average response in the stimulus period was less than the average baseline ITI response. To determine if a visually-responsive neuron was sensitive to a particular level of scrambling, we compared the responses to the four objects for each of the five scrambling levels using a one-way AVOVA (*p* < 0.05). Neurons with a significant effect of scrambling were then assessed using a Tukey Honest Significant Difference post-hoc comparison test (*p* < 0.05) to determine which levels of scrambling the neuron was responding to.

### Population data analysis

So that cells with different levels of activity across the time course of the stimulus period can be compared fairly, the firing rate of each cell in the stimulus period (1.5 s) was normalised relative to the average firing rate during a corresponding window in the middle of the baseline ITI (1.5 s). The dependence of normalised firing rate for each stimulus on the horizontal spatial frequency, is then extracted with a Bayesian linear model. This is used to produce a total posterior distribution of the spatial frequency dependence of the firing rates in each region (the slopes of the linear model) as shown in Fig. 4. To compare these distributions and demonstrate the differences between regions, we use the Bayesian estimation test of Kruschke^18^. Briefly, we treat the distribution of spatial frequency dependence for each region as a Student-t distribution, which extends a Gaussian distribution with the potential for heavy tails. The means and standard deviations for each neural region are considered to be sampled from the same distributions in a hierarchical model. The relative height of the tails of the estimated t-distributions for each group is denoted by the Greek letter *ν* (nu)*.* A small *ν* value reflects a t distribution with heavy tails, and a large *ν* value (e.g., 100) reflects a t distribution that is nearly normal^18^. The prior distributions used in all Bayesian calculations are flat or effectively flat. This modelling is performed in Python 3 using the pyMC3 package, which provide estimates of the posterior distributions using Markov-chain Monte-Carlo methods.

## Supplementary Information

Supplementary Information.

## Data Availability

The datasets supporting the findings of this study have been uploaded as part of the supplementary material and are available at Mendeley Data: Clark, William; Chilcott, Matthew; Colombo, Michael (2022), “The effect of progressive image scrambling on neuronal responses at three stations of the pigeon tectofugal pathway”, Mendeley Data, V1, https://doi.org/10.17632/mwswh4rys6.2.

## Code availability

The original code supporting the findings of this study have been uploaded as part of the supplementary material and are available at Mendeley Data: Clark, William; Chilcott, Matthew; Colombo, Michael (2022), “The effect of progressive image scrambling on neuronal responses at three stations of the pigeon tectofugal pathway”, Mendeley Data, V1, https://doi.org/10.17632/mwswh4rys6.2.
