# Supplementary figures and images for: The effect of progressive image scrambling on neuronal responses at three stations of the pigeon tectofugal pathway
Source: Sci Rep. 2022 Aug 19;12:14190. doi: 10.1038/s41598-022-18006-0 (PMC9391454; doi:10.1038/s41598-022-18006-0)

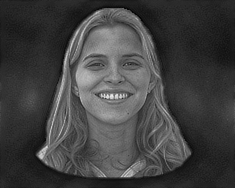

Supplement: Supplementary file 1 — Supplementary Information. [file 41598_2022_18006_MOESM1_ESM.zip › Supporting data and code/Spectral analysis code, data, and image set/stimuli/NAT1_0.gif]

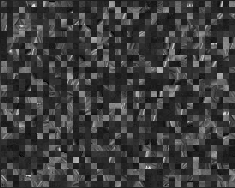

Supplement: Supplementary file 1 — Supplementary Information. [file 41598_2022_18006_MOESM1_ESM.zip › Supporting data and code/Spectral analysis code, data, and image set/stimuli/NAT1_1209.gif]

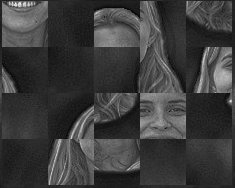

Supplement: Supplementary file 1 — Supplementary Information. [file 41598_2022_18006_MOESM1_ESM.zip › Supporting data and code/Spectral analysis code, data, and image set/stimuli/NAT1_20.gif]

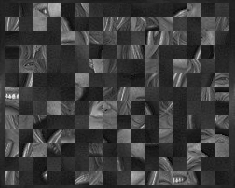

Supplement: Supplementary file 1 — Supplementary Information. [file 41598_2022_18006_MOESM1_ESM.zip › Supporting data and code/Spectral analysis code, data, and image set/stimuli/NAT1_208.gif]

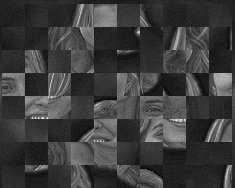

Supplement: Supplementary file 1 — Supplementary Information. [file 41598_2022_18006_MOESM1_ESM.zip › Supporting data and code/Spectral analysis code, data, and image set/stimuli/NAT1_80.gif]

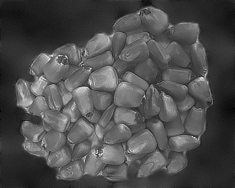

Supplement: Supplementary file 1 — Supplementary Information. [file 41598_2022_18006_MOESM1_ESM.zip › Supporting data and code/Spectral analysis code, data, and image set/stimuli/NAT2_0.gif]

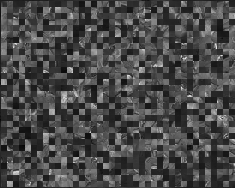

Supplement: Supplementary file 1 — Supplementary Information. [file 41598_2022_18006_MOESM1_ESM.zip › Supporting data and code/Spectral analysis code, data, and image set/stimuli/NAT2_1209.gif]

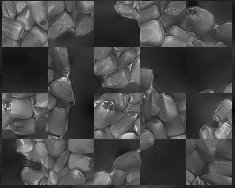

Supplement: Supplementary file 1 — Supplementary Information. [file 41598_2022_18006_MOESM1_ESM.zip › Supporting data and code/Spectral analysis code, data, and image set/stimuli/NAT2_20.gif]

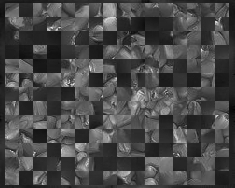

Supplement: Supplementary file 1 — Supplementary Information. [file 41598_2022_18006_MOESM1_ESM.zip › Supporting data and code/Spectral analysis code, data, and image set/stimuli/NAT2_208.gif]

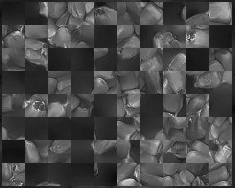

Supplement: Supplementary file 1 — Supplementary Information. [file 41598_2022_18006_MOESM1_ESM.zip › Supporting data and code/Spectral analysis code, data, and image set/stimuli/NAT2_80.gif]

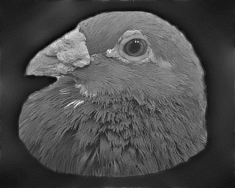

Supplement: Supplementary file 1 — Supplementary Information. [file 41598_2022_18006_MOESM1_ESM.zip › Supporting data and code/Spectral analysis code, data, and image set/stimuli/NAT3_0.gif]

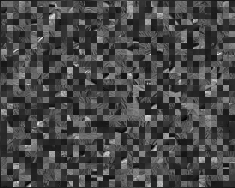

Supplement: Supplementary file 1 — Supplementary Information. [file 41598_2022_18006_MOESM1_ESM.zip › Supporting data and code/Spectral analysis code, data, and image set/stimuli/NAT3_1209.gif]

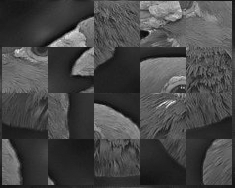

Supplement: Supplementary file 1 — Supplementary Information. [file 41598_2022_18006_MOESM1_ESM.zip › Supporting data and code/Spectral analysis code, data, and image set/stimuli/NAT3_20.gif]

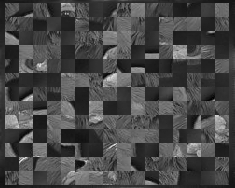

Supplement: Supplementary file 1 — Supplementary Information. [file 41598_2022_18006_MOESM1_ESM.zip › Supporting data and code/Spectral analysis code, data, and image set/stimuli/NAT3_208.gif]

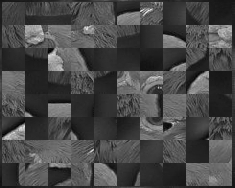

Supplement: Supplementary file 1 — Supplementary Information. [file 41598_2022_18006_MOESM1_ESM.zip › Supporting data and code/Spectral analysis code, data, and image set/stimuli/NAT3_80.gif]

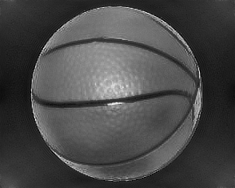

Supplement: Supplementary file 1 — Supplementary Information. [file 41598_2022_18006_MOESM1_ESM.zip › Supporting data and code/Spectral analysis code, data, and image set/stimuli/NAT4_0.gif]

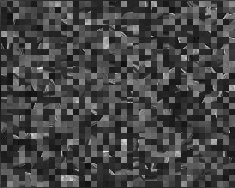

Supplement: Supplementary file 1 — Supplementary Information. [file 41598_2022_18006_MOESM1_ESM.zip › Supporting data and code/Spectral analysis code, data, and image set/stimuli/NAT4_1209.gif]

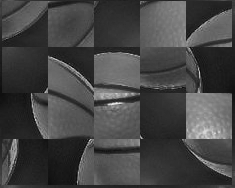

Supplement: Supplementary file 1 — Supplementary Information. [file 41598_2022_18006_MOESM1_ESM.zip › Supporting data and code/Spectral analysis code, data, and image set/stimuli/NAT4_20.gif]

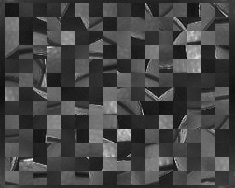

Supplement: Supplementary file 1 — Supplementary Information. [file 41598_2022_18006_MOESM1_ESM.zip › Supporting data and code/Spectral analysis code, data, and image set/stimuli/NAT4_208.gif]

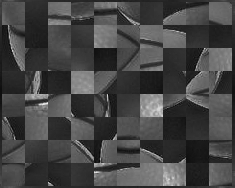

Supplement: Supplementary file 1 — Supplementary Information. [file 41598_2022_18006_MOESM1_ESM.zip › Supporting data and code/Spectral analysis code, data, and image set/stimuli/NAT4_80.gif]
